# Long-term patient-reported back and shoulder function after delayed breast reconstruction with a latissimus dorsi flap: case–control cohort study

**DOI:** 10.1093/bjs/znad296

**Published:** 2023-10-25

**Authors:** Jonas Löfstrand, Anna Paganini, Anna Grimby-Ekman, Mattias Lidén, Emma Hansson

**Affiliations:** Department of Plastic Surgery, Institute of Clinical Sciences, The Sahlgrenska Academy, University of Gothenburg, Gothenburg, Sweden; Department of Plastic Surgery, Region Västra Götaland, Sahlgrenska University Hospital, Gothenburg, Sweden; Department of Plastic Surgery, Institute of Clinical Sciences, The Sahlgrenska Academy, University of Gothenburg, Gothenburg, Sweden; Department of Plastic Surgery, Region Västra Götaland, Sahlgrenska University Hospital, Gothenburg, Sweden; Institute of Health and Care Sciences, Sahlgrenska Academy, University of Gothenburg, Gothenburg, Sweden; School of Public Health and Community Medicine, Institute of Medicine, The Sahlgrenska Academy, University of Gothenburg, Gothenburg, Sweden; Department of Plastic Surgery, Institute of Clinical Sciences, The Sahlgrenska Academy, University of Gothenburg, Gothenburg, Sweden; Department of Plastic Surgery, Region Västra Götaland, Sahlgrenska University Hospital, Gothenburg, Sweden; Department of Plastic Surgery, Institute of Clinical Sciences, The Sahlgrenska Academy, University of Gothenburg, Gothenburg, Sweden; Department of Plastic Surgery, Region Västra Götaland, Sahlgrenska University Hospital, Gothenburg, Sweden

## Abstract

**Background:**

Sacrifice of the latissimus dorsi (LD) muscle might entail donor site morbidity when used in delayed breast reconstruction. Previous studies are small, have short follow-up, and demonstrate diverging results. The aims of this study were to evaluate long-term patient-reported effects on shoulder and back function following LD flap harvest, and to investigate predictors for a worse outcome.

**Method:**

This is a retrospective observational case–control cohort study. Cases were all patients who had undergone an LD flap reconstruction during the years 2007–2017. Controls were patients reconstructed with a deep inferior epigastric perforator (DIEP) flap during the same time period. Participants completed two validated questionnaires; the BREAST-Q reconstruction LD domains and the Western Ontario Shoulder Osteoarthritis Index (WOOS).

**Results:**

A total of 135 cases (75 per cent) and 118 controls (60 per cent) responded to the questionnaires. The mean follow-up time was 7 years. Patients reconstructed with a LD flap were significantly less satisfied with their back and shoulder function when compared to the DIEP controls, as measured with BREAST-Q and WOOS. Predictors for a poor patient-reported back and shoulder function included axillary surgery and axillary radiotherapy, especially when combined, as well as higher age at reconstruction.

**Conclusion:**

Patients who have undergone LD flap for delayed breast reconstruction had a lower satisfaction with back and shoulder function, when compared to patients who had undergone a DIEP reconstruction. Delayed LD reconstruction should be used with care, especially in patients who have undergone axillary surgery and axillary radiotherapy.

## Teaser

This is a retrospective observational case–control study, evaluating patient reported back and shoulder function 7 years after a breast reconstruction with latissimus dorsi reconstruction. Women with similiar age, BMI and radiation status were used as controls. The patients were statistically significantly less satisfied with their back and shoulder function when compared to the controls, as measured with BREAST-Q and WOOS. Axillary surgery was the only predictor that was statistically significantly associated with low satisfaction.

## Introduction

Breast reconstruction after mastectomy is an integral part of modern breast cancer treatment. Many defects can be reconstructed by means of expander- and implant-based methods, but some defects, especially in the irradiated chest wall, require autologous tissue transfer. The most commonly used options include abdominal free flaps^1^, such as the deep inferior epigastric perforator (DIEP) and the superficial inferior epigastric artery (SIEA) flaps, or pedicled flaps from the back, such as the latissimus dorsi (LD) and the thoracodorsal artery perforator (TDAP) flaps^2^. Abdominal free flaps might not be suitable when a patient has limited abdominal adipose tissue, extensive abdominal scarring, a desire to avoid an abdominal donor site scar, or when microsurgical technique is unsuitable due to other reasons such as co-morbidities. In such patients a pedicled flap from the back, most commonly the LD flap, is a viable option.

The use of the LD flap was first introduced by Tanzini in 1897^3^ for the coverage of a mastectomy defect. Several decades later, in 1976, Olivari^4^ reintroduced, and subsequently popularized, the technique for use in previously irradiated patients. The technique has been further refined and used in combination with implants^5^, with a skin paddle^6^, as a muscle-sparing flap^7^, as a buried flap^8^ and as an extended flap in which extra fat is included to create more bulk^9^. The LD flap has historically been a commonly used flap for breast reconstruction, but with the introduction and popularization of the DIEP flap, it is less used today. This shift is also evident at the authors’ unit. However, during the study period the use of the two flaps was nearly equal.

The LD muscle is the largest muscle in the torso and has several functions: extension and adduction of the humerus as well as internal rotation of the shoulder. Hence, the sacrifice of such a muscle raises concern of donor site morbidity. There are two systematic reviews and meta-analyses on donor-site morbidity after LD flap transfer^10,11^. Both focus on donor-site morbidity, mainly shoulder function. Steffenssen *et al.*^10^ included 26 articles evaluating shoulder range of motion (ROM), shoulder strength, subjective discomfort symptoms and the Disabilities of the Arm, Shoulder, and Hand Questionnaire (DASH) score. Lee and Mun^11^ included 22 articles (644 patients) that evaluated the outcomes shoulder ROM, shoulder strength, DASH score and subjective evaluation. Both conclude that most patients experience limited impairment in shoulder function, which over time recovers to near normal. Some patients, however, have significant reduction in shoulder function and strength in the early postoperative phase and sometimes long term^11^, and little is known about why^10^. Few previous studies have controlled for confounding factors that may affect back and shoulder function, such as age, type of LD flap, axillary surgery, radiotherapy, mastectomy and physiotherapy^10^. In addition, most studies had short follow-up and sample sizes were generally small^10,11^.

Regarding controlled studies, a prospective study comparing 40 patients who had received an LD flap for immediate breast reconstruction with 44 patients who had been operated with mastectomy alone concluded that both groups had similar reductions in shoulder function, as measured with shoulder ROM, one year after the operation^12,13^. Similarly, a study comparing 35 patients reconstructed with LD flaps plus implants with 41 patients reconstructed with implants alone concluded that the patient-reported shoulder function was similar in both groups, although slightly worse in the implant group (follow-up 1–10 years). These women had all undergone mastectomy, axillary surgery and radiotherapy^14^. However, irradiated patients reconstructed with implants only might be an unsuitable control group, because it involves pectoralis major muscle division and is prone to radiation-induced capsular contracture potentially affecting shoulder mobility^14^.

The primary aim of this study was to investigate long-term effects of LD flap transfer on patient-reported shoulder and back function compared to a control group consisting of similar patients reconstructed with a DIEP flap. The secondary aim was to examine predictors for a poor patient-reported back and shoulder function.

## Methods

### Study design and protocol

This study is a retrospective observational case–control cohort study, reported according to the STROBE recommendations. It is one of the studies described in the ‘Reconstruction with back donor site flaps study’ protocol (ClinicalTrials.Gov identifier NCT04526561). Some of the data have previously been included in a validation study^15^.

### Ethics and informed consent

The Regional Ethical Committee of Gothenburg/the Swedish Ethical Review Authority reviewed and approved the study (254-18, 2021-00432 and 2022-06235-02). Procedures followed were in accordance with the Helsinki Declaration and Good Clinical Practice (GCP) guidelines. All participants gave their informed consent to participate.

### Setting, patients and controls

The study was performed in the Department of Plastic and Reconstructive Surgery, Sahlgrenska University Hospital, in Sweden. The department annually performs approximately 350 breast reconstructions, including both immediate and delayed reconstructions. According to Swedish national guidelines^16^, autologous breast reconstruction is offered principally to patients who have had previous radiotherapy. When both options are technically possible, these patients are informed of both DIEP and LD flap techniques, and thereafter choose the reconstruction method they prefer^16^. Patients who are not candidates for a DIEP flap, due to excessive scarring of the abdomen, scarce abdominal body fat or co-morbidities, are offered the LD flap. The LD flap may also be used where previous reconstruction methods have failed as a salvage LD flap^16^. To be eligible for breast reconstruction the patient must have a BMI ≤30, abstain from smoking at least 6 weeks prior and after the operation, as well as have no signs of generalized breast cancer.

All consecutive patients operated with a delayed LD flap breast reconstruction following mastectomy and radiotherapy during 2007–2017 were offered inclusion. Patients operated consecutively during the same period with a DIEP flap after mastectomy and radiotherapy were recruited as controls. All patients and controls were identified through the operation planning system. DIEP patients were chosen as controls as they, similarly to the LD group, had undergone axillary surgery and radiotherapy and were of a similar age, thus allowing control for factors that might affect shoulder and back function^10,11^. All patients had a BMI of <30, were non-smokers at the time of operation and underwent a delayed breast reconstruction. During a part of the study period, patients were included in the GoBreast study (ClinicalTrials.Gov identifier NCT03963427)^17^.

### Exposure

Cases were operated with a LD flap and controls with a DIEP flap^1^, and the authors’ department has extensive experience using both methods^18–21^. All LD flaps were classical pedicled musculocutaneous, non-extended, non–muscle-sparing flaps^6^, combined with a pre-pectoral implant and performed as a delayed reconstruction. Implants used in this study were anatomical silicone implants (CPG™ Gel Breast Implants, Cohesive III™, Mentor, Irvine, CA, USA) and expanders temporary (CPX™, Mentor) or permanent (Siltex™ Becker 35, Mentor).

### Outcomes and clinical variables

In September 2020–June 2021, patients were sent an envelope including information regarding the study, the questionnaires, a consent form and a stamped return envelope. Two reminders were sent after 3 and 5 weeks, respectively. Two questionnaires were used; the BREAST-Q reconstruction LD domains^22^ and the Western Ontario Shoulder Osteoarthritis Index (WOOS)^23^. BREAST-Q was used under licence from Memorial Sloan Kettering Cancer Center, New York, USA. WOOS was used by permissions from researchers who had performed a Swedish validation.

The BREAST-Q LD reconstruction questionnaire has two domains concerning satisfaction with donor site after LD harvest. These are ‘satisfaction with back appearance’ (8 items) and ‘satisfaction with back and shoulder function’ (11 items), where patients rate how often they have been bothered by different problems, during the last 2 weeks, on a 5-point scale ranging from ‘none of the time’ to ‘all of the time’^22^. Sum scores of the two domains are converted to a score between 0 and 100, where a higher score represents a lower symptom burden^22,24,25^. These conversion tables have been created based on Rasch transformation of the total raw scores, which have then been logarithmized to fit into the 0–100 format. The questionnaire has been translated to Swedish and validated in a Swedish population^15^. There are no published minimal clinically important differences (MCIDs) for BREAST-Q LD. Previous studies^22,26^ using BREAST-Q LD are summarized in the *Material S1*.

The WOOS^23^ is a 19-item scale with four domains: physical symptoms and pain (six items), sport, recreation, and work (five items), lifestyle and social functioning (five items), and emotional well-being (three items). The patient indicates his/her function in each item on a horizontal visual analogue scale (VAS) from 0 to 100 mm, where each millimetre is equal to one point. VAS scores are generally considered ordinal scales^27^. A sum score is calculated for each domain, where a higher score indicates a higher symptom burden. A total score is also calculated, where 0 points represent no deficit and 1900 points the highest symptom burden possible. The scores are presented as a percentage of the score of a healthy shoulder, were 100 per cent represents a shoulder with perfect function. The questionnaire has been translated and cross-culturally validated for Swedish^28^. Anchor-based MCID for the WOOS total, calculated in patients with glenohumeral osteoarthritis treated with an anatomical total shoulder arthroplasty, is 12.3 percentage points^29^. Examples of previous studies^29,30^ using WOOS are given in *Material S1*.

Clinical data were collected from medical charts and included age at time of reconstruction, years since reconstructive surgery, previous radiotherapy and type of radiotherapy (subdivided into targeted to the chest wall or axilla, or both), use of aromatase inhibitors, unilateral/bilateral operation, type of axillary surgery (sentinel node biopsy or axillary dissection), denervation of the thoracodorsal nerve (primary denervation when the flap was raised or secondary denervation in a subsequent operation), one- or two-stage reconstruction, postoperative complications and need of corrective surgery. Postoperative breast-specific complications were categorized into seroma, infection, bleeding, wound healing problems or removal of implant. Seromas were only registered if they needed puncture. Infection was registered whenever patients were prescribed antibiotics after the initial peri- and postoperative treatment. Corrective surgery included correction of implant placement, capsulectomy, implant change, lipofilling and skin correction. Complications of contralateral surgery on healthy breasts were not registered.

### Statistics

Statistical analyses were preformed using Excel® version 16.30 (Microsoft Corporation, Redmond, Washington, USA), QScore^TM^ (the Mapi Research Trust, Lyon, France), and SPSS® version 27.0.0.0 (IBM, Armonk, New York, USA) and SAS® v 9.3 (SAS Institute, Cary, North Carolina, USA). BREAST-Q was scored with QScore^TM^. If at least half of the items of a BREAST-Q domain were answered, missing data were replaced with the mean of the answered items for each domain. The domain was excluded if more than half of the items were missing. Domains of WOOS with missing data were excluded. Ordinal data were presented as medians, and ranges and continuous data as means and s.d.s. BREAST-Q and WOOS scores were illustrated in radar charts. Mann–Whitney U-test was used to compare BREAST-Q and WOOS percentage of normal shoulder scores. To investigate predictors for a low patient-reported function (outcomes: BREAST-Q back and shoulder function scores and WOOS -percentage scores) regression analysis was performed. The potential predictors investigated were age at time of surgery, years after reconstruction, axillary surgery (sentinel node biopsy/axillary dissection/missing), use of aromatase inhibitors (yes/no), radiotherapy (chest wall/chest wall and axilla) and complication within the first 30 days (yes/no). Bilateral flaps were not included as a potential predictor as there were only eight patients who underwent such operations. Denervation was not included as it had many missing values. The linear regression was preceded by a collinearity check, using paired Spearman correlation of potential predictors. Linear regression was performed with BREAST-Q back and shoulder function scores and WOOS percentage scores as dependent variables and the potential predictors as independent variables. For each outcome a regression model was fitted including the predictors age at time of reconstruction, years after reconstruction, axillary surgery, radiotherapy target, complications within 30 days and usage of aromatase inhibitors. Axillary surgery and radiotherapy given to both the chest wall and the axilla were closely linked. Therefore, one model including a predictor with the three categories axillary surgery, axillary dissection with radiotherapy to the chest wall and axillary dissection with radiotherapy to the chest wall and the axilla was performed (model 1, *Table 3*), and another model with the radiotherapy target (model 2, *Table 3*). Residuals for each regression analysis were checked for the assumptions of normal distribution and constant deviation along the predicted values, and no strong deviations from these assumptions were seen. All tests were two-tailed and *P* ≤ 0.05 was considered to indicate a statistical significance.

**Table 1:** Demographics and surgical details

	LD (cases) (*n* = 135 patients, 143 flaps)	DIEP (controls) (*n* = 118 patients, 126 flaps)
**Age at reconstruction** (median (range), mean (s.d.))	53 (32–75) 52 (9.7)	50 (33–66) 50 (7)
**Age at time of questionnaire** (median (range), mean (s.d.))	60 (38–81) 59 (10)	58 (43–76) 59 (7)
**Years since reconstruction** (median (range), mean (s.d.))	7 (3–11) 7 (2)	7 (3–14) 7 (3)
**Radiotherapy**		
Target chest wall	58	42
Target both chest wall and axilla	64	63
Radiotherapy given, but target unknown	3	4
No radiotherapy	10	9
**Use of aromatase inhibitors (*n*)**		
Yes	47	35
No	85	80
Data missing	3	3
**Axillary surgery (*n*, %)**		
Sentinel node biopsy	22 (16%)	14 (12%)
Axillary dissection	83 (62%)	101 (86%)
Data missing	30 (22%)	3 (3%)
**Number of flaps (*n*, %)**		
Unilateral	127 (89%)	110 (87%)
Bilateral	16 (11%)	16 (13%)
**Indication for flap reconstruction (*n*, %)**		
Previous radiotherapy	124	115
Salvage	11	8*
Previous implant-based reconstruction	5	5
Randomized in GoBreast	3	10
**Implant/stages (*n*, %)**		
Two-stage (expander + implant)	28 (21%)	
One-stage (implant)	106 (79%)	
Autologous LD	0	
Data missing	1 (1%)	
**Denervation (*n*, %)**		
None	15 (11%)	
Primary	33 (24%)	
Secondary	3 (2%)	
Data missing	84 (62%)	
**Re-operation due to complications**† **(*n*, %)**		
Yes (*n*, %)	15 (11%)	22 (19%)
No (*n*, %)	120 (89%)	96 (81%)
Donor site haematoma	0	0
Breast haematoma	3 (2%)	5 (4%)
Removal of implant	7 (5%)	–
Anastomosis insufficiency	–	4 (3%)
Wound dehiscence/breast necrosis	5 (4%)	20 (17%)
Wound dehiscence/donor site necrosis	0	4 (3%)
**Complications not requiring surgery (*n*, %)**		
Donor site seroma	18 (13%)	2 (2%)
Breast seroma	0	
Donor site infection	6 (4%)	14 (12%)
Breast infection	10 (7%)	25 (21%)
Wound dehiscence at donor site	20 (15%)	18 (15%)
Wound dehiscence breast	5 (4%)	33 (28%)
Bulging	**–**	4 (3%)
**Surgical corrections at donor site (*n*, %)**		
Repair of abdominal wall hernia	0	1 (1%)
Skin correction	0	24 (20%)
Liposuction	0	2 (2%)
Umbilicoplasty	**–**	1 (1%)
**Surgical corrections breast**‡ **(*n*, %)**		
Yes	50 (37%)	53 (45%)
No	85 (63%)	65 (55%)
Capsulectomy/adjustment of implant location	16 (12%)	**–**
Implant size change	8 (6%)	
Lipofilling	27 (20%)	19 (16%)
Skin correction	11 (8%)	10 (8%)
Trimming of flap	0	25 (21%)
Liposuction	0	14 (12%)
Implant insertion	–	1 (1%)

^*^Three of the salvage patients had undergone radiation and are therefore counted in both groups. †Complications occurring within 30 days after surgery. Systemic complications such as pulmonary embolism and sepsis not registered. ‡Some patients had more than one complication.

**Table 2:** Patient-reported back and shoulder function

	LD			DIEP			Average difference in points between LD and DIEP	Statistical difference between LD and DIEP (*P*)
	Unilateral	Bilateral	Total	Unilateral	Bilateral	Total		
**BREAST-Q (0–100)**	(*n* = 127)	(*n* = 8)	(*n* = 135)	(*n* = 109)	(*n* = 9)	(*n* = 118)		Mann-Whitney U-test
Satisfaction with shoulder and back function (median (range), mean (s.d.))	59 (0–100) 63 (21)	58 (26–100) 60 (27)	58 (0–100) 63 (21)	67 (21–100) 70 (23)	57 (49–100) 70 (21)	66 (21–100) 70 (23)		0.006
**WOOS**	(*n* = 126)	(*n* = 6)	(*n* = 132)	(*n* = 108)	(*n* = 8)	(*n* = 116)		
**Total score (0–1900)** (median (range))								
	248 (61–1755)	218 (60–1110)	241 (60–1755)	133 (11–1721)	192 (86–603)	(11–1721)		
**Per cent of normal function** (median (range), mean (s.d.))								
Total	87 (8–97)	89 (42–97)	87 (8–97)	93 (9–99)	90 (68–95)	93 (9–99)	Total: 6 Unilateral: 6 Bilateral: 1	0.02
Physical symptoms	89 (5–97)	92 (52–96)	89 (5–97)	94 (9–100)	91 (86–97)	94 (9–100)	Total: 5	<0.001
Sports/recreation/work	86 (8–97)	71 (29–97)	86 (8–97)	93 (4–100)	89 (37–96)	93 (4–100)	Total: 7	0.001
Lifestyle	88 (9–99)	87 (15–97)	87 (9–99)	93 (5–100)	90 (77–95)	92 (5–100)	Total: 5	0.014
Emotions	93 (10–99)	96 (52–97)	93 (10–99)	94 (11–100)	93 (71–95)	94 (11–100)	Total: 1	0.754

**Table 3:** Linear regression of low satisfaction measured with BREAST-Q and WOOS as the dependent variables

Predictors	Model 1∗ *n* = 102			Model 2† *n* = 122		
	Coefficient	95% c.i.	*P*	Coefficient	95% c.i.	*P*
**BREAST-Q as dependent variable**						
Intercept	40.2	23.19; 57.17	<0.001	35.1	20.11; 50.09	<0.001
Age at reconstruction	0.04	0.209; 0.296	0.733	0.11	−0.117; 0.328	0.351
Years after reconstruction	−0.69	−1.74; 0.350	0.190	−0.39	−1.30; 0.52	0.396
Axillary surgery	7.7	2.05;13.28	0.014			
Axillary surgery and radiotherapy to the breast	5.4	0.090; 10.64				
* Ref. Clearance and radiotherapy to the chest wall and the axilla* Use of aromatase inhibitors	−0.28	−5.25; 4.69	0.912	0.96	−3.326; 5.242	0.659
Complication during the 30 first days	−0.65	−5.24; 3.95	0.780	−0.66	−4.755; 3.426	0.748
Radiotherapy target *Ref. Chest wall and axilla*				4.7	0.81; 8.53	0.018
*R*^2^	0.10			0.06		
Adj *R*^2^	0.04			0.02		
**WOOS % as dependent variable**	**Model 1** ***n* = 99**			**Model 2** ***n* = 119**		
Intercept	70.0	39.17; 100.8	<0.001	35.1	20.11; 50.09	<0.001
Age at reconstruction	0.34	−0.120; 0.803	0.145	0.11	−0.117; 0.328	0.351
Years after reconstruction	−1.75	−3.62; 0.114	0.065	−0.39	1.30; 0.52	0.396
Axillary surgery	7.08	−3.23; 17.40	0.318			
Axillary surgery and radiotherapy to the breast *Ref. Dissection and chest wall and axillary radiation*	5.06	−4.42–14;54				
Use of aromatase inhibitors	3.6	−5.50; 12.69	0.434	3.8	−3.74; 11.29	0.322
Complication during the 30 first days	−4.1	12.38; 4.22	0.331	−4.07	11.18; 3.04	0.259
Radiotherapy target *Ref. Chest wall and axilla*				4.9	−1.83; 11.68	0.152
*R*^2^	0.10			0.06		
Adj. *R*^2^	0.04			0.02		

∗Model 1 includes a predictor with three categories: axillary surgery, axillary dissection with chest wall radiation and axillary dissection with chest wall and axillary radiation. †Model 2 includes the category radiotherapy target (chest wall/chest wall + axilla) as a predictor.

## Results

### Participants

**Fig. 1:**
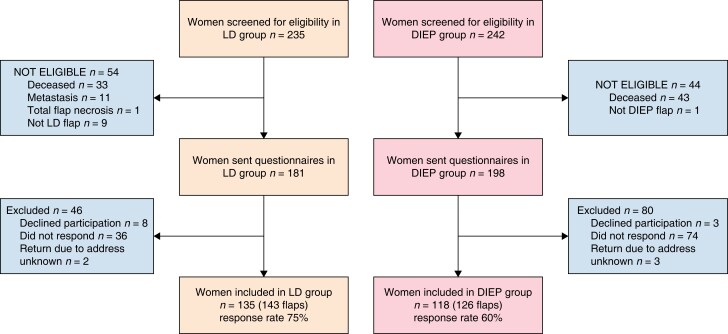
STROBE diagram

During 2007–2017, 226 patients had a delayed breast reconstruction with an LD flap (cases) and 241 with a DIEP flap (controls). When the study was performed, mortality rates in the two groups were 15 (*n* = 33) and 18 (*n* = 43) per cent, respectively. Among eligible patients, 75 per cent of the LD patients and 60 per cent of the control group responded to the questionnaires (*Fig. 1*). Age, follow-up time, axillary surgery and radiotherapy were similar in the two groups. *Table 1* shows demographic and clinical details, including complications and re-operations.

### Patient reported back and shoulder function

**Fig. 2:**
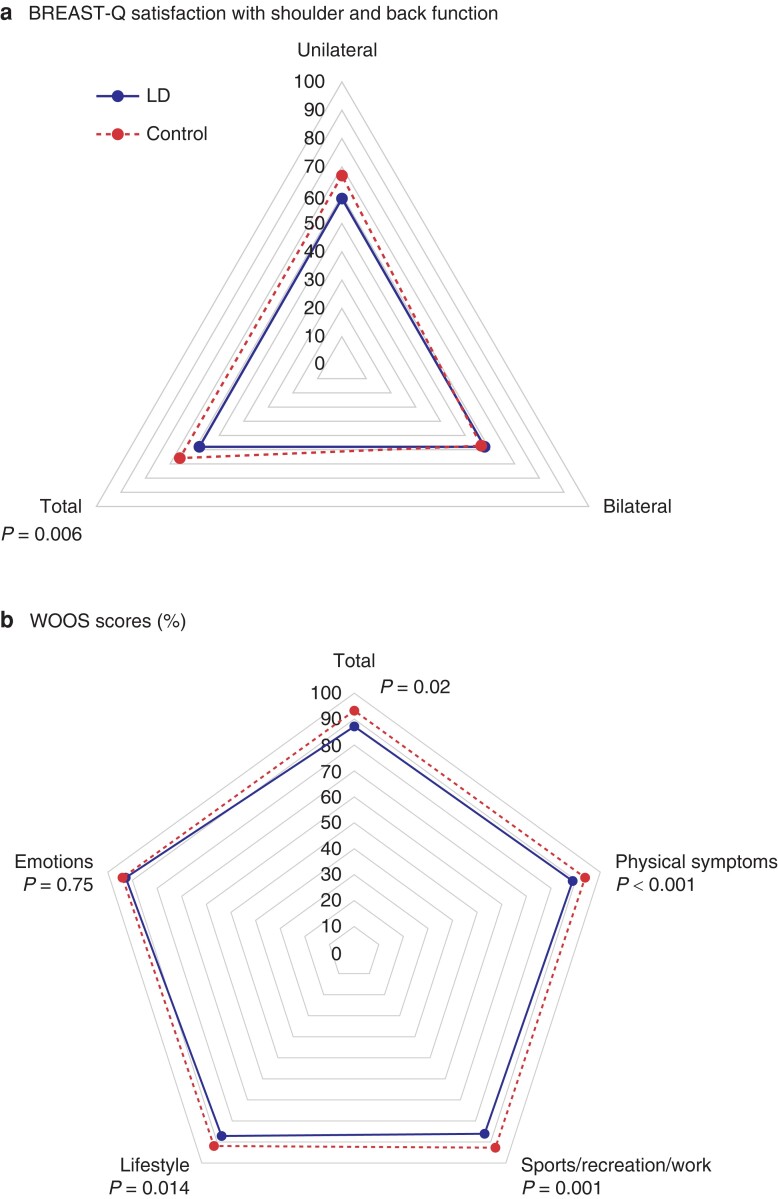
**a** Radar chart of median BREAST-Q reconstruction scores for the domain ‘back and shoulder function’. Mann–Whitney U-test was used to compare LD cases and DIEP controls. Scores ranged from 0 to 100 for cases and 21 to 100 for controls. Mean score for LD cases were 63 (s.d. 21) and 70 (s.d. 23) for DIEP controls. **b** Radar chart of median WOOS scores (per cent of normal function) in total and for different domains. Mann–Whitney U-test was used to compare cases and controls. Total scores ranged from 8 to 97 per cent for LD cases and from 4 to 100 for DIEP controls

**Fig. 3:**
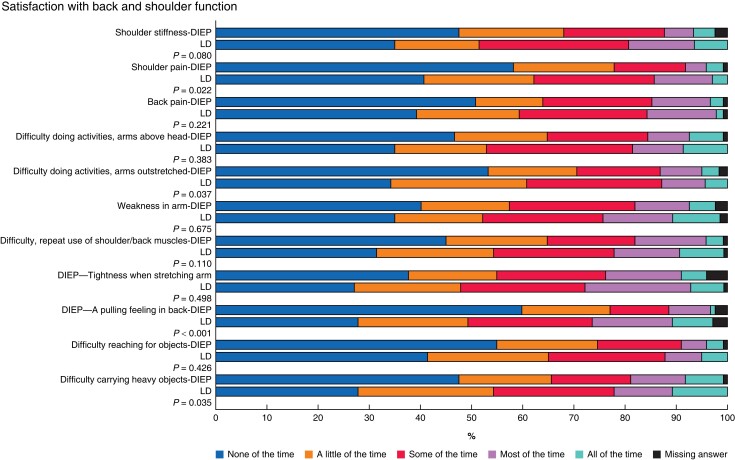
Frequency of answers for different items in BREAST-Q back and shoulder function

According to BREAST-Q, the LD patients were less satisfied with their back and shoulder function compared to the DIEP controls (*P* = 0.006) (*Table 2* and *Fig. 2*). According to WOOS, the LD patients had a lower function than the controls, both in terms of total function (median score 248 (range 60–1755) *versus* 139 (11–1761)) as in subdomains (*Table 2* and *Fig. 2*). Although the differences were statistically significant in all domains, except ‘emotions’, they were, however, considerably smaller than the MCID (12.3 percentage points)^29^. The number of patients who had back pain most/all of the time was 15 per cent in the LD group and 14 per cent in the DIEP control group, whereas and shoulder pain most/all of time were 14 and 7 per cent, respectively (*Fig. 3*). Symptoms that more than 20 per cent of the LD patients experienced most/all of the time were: tightness when stretching arm (27 per cent *versus* 20 per cent compared to DIEP controls), pulling feeling in back (24 per cent *versus* 9 per cent), weakness in arm (23 per cent *versus* 16 per cent), difficulty carrying heavy objects (22 per cent *versus* 18 per cent) and difficulty of repeat shoulder/back muscle use (21 per cent *versus* 17 per cent) (*Fig. 3*). Regarding symptoms, there was a statistically significant difference between the groups for shoulder pain, difficulty doing activities arms outstretched, a pulling feeling in back and difficulty carrying heavy objects (*Fig. 3*).

### Predictors for a low back and shoulder function

The only statistically significant predictor for a low satisfaction measured with BREAST-Q was the combination of axillary surgery and type of radiation (*P* = 0.014), with an estimated difference of 7.7 (95 per cent c.i. 2.05; 13.28) between sentinel node biopsy and axillary dissection in combination with chest wall and axillary radiotherapy (Model 1 in *Table 3*), and chest wall and axillary radiotherapy (*P* = 0.018) with an estimated difference of 4.7 (95 per cent c.i. 0.81; 8.53; Model 2 in *Table 3*). Measured with WOOS%, the only statistically significant predictor was age at reconstruction (*P* = 0.035), where older age at reconstruction was associated with a higher symptom burden.

## Discussion

This is a study evaluating patient-reported satisfaction of back and shoulder function in patients who have undergone a delayed breast reconstruction, following mastectomy and radiotherapy, with either an LD flap (cases) or a DIEP flap (controls) with a mean follow-up time of 7 years and using validated questionnaires. The results show that those operated with LD flaps have lower back and shoulder function than the DIEP controls. The only statistically significant predictors for a low satisfaction were axillary surgery (*P* = 0.014), axillary radiotherapy (*P* = 0.018) and age at reconstruction (*P* = 0.035).

The retrospective design of the study results in several limitations, most notably missing data regarding clinical factors that might affect the function after surgery, such as denervation of the muscle during the primary surgery, history of shoulder and back disease, physiotherapist treatment, current BMI and smoking status and knowledge about preoperative satisfaction with back and shoulder function^10^. Moreover, as the groups were operated over a period of 10 years by different surgeons, there might have been slight variations in the surgical techniques that could affect the effect on back and shoulder function. Other study design aspects that might have affected the results is that only patients operated on with an LD in combination with an implant were included, and that outcomes were limited to patient-reported outcomes. The study could have been strengthened if a combination of patient-reported outcomes and objectives measures of shoulder and back function were used. However, it was not feasible to perform objective measurements in the current study.

Another limitation is that an analysis of non-responders was not possible as they did not consent to chart review. Although response rates were 77 and 60 per cent, respectively, and are above that expected at 7 years after surgery^31^, the imperfect response rates could have introduced a bias, as could the different response rates between the groups. In the present study, it was clear in the information presented to the participants that the aim was to compare back and should function after an LD flap with controls, and therefore the DIEP flap controls might have been less motivated to participate. Compared to the present sample sizes, the most recent systematic reviews^10,11^ included studies with a maximum sample of 73 (6-month follow-up)^32^ and 121 patients (22-month follow-up)^33^, respectively. Both studies^32,33^ used physical examination as the evaluation method. The included studies using validated questionnaires have samples of between 12 and 58 patients^10,11^. This is in contrast to the current study with 140 patients and a mean/median follow-up of 7 years.

The inclusion of a control group and the homogeneity of the two groups regarding mastectomy, radiotherapy and axillary surgery, three factors that themselves may affect shoulder function^34–37^, increases the probability that the actual effect of an LD flap is measured. Nonetheless, the homogeneity of the groups might be affected by the choice of reconstructive method given to the patient. Potential negative effects on shoulder function of an LD flap is discussed with the patients before surgery and there might be a tendency that patients already suffering from shoulder and back dysfunction after breast cancer surgery^34–39^ choose a DIEP flap instead of an LD flap. If this is the case, the actual difference in shoulder function between the studied groups might be even larger than reported here.

Previous studies on patient-reported back and shoulder functions have typically used the DASH questionnaire, which measures physical function and symptoms of the entire upper extremity^10^. Therefore, it includes a number of items that are not relevant to evaluate the effect of an LD transfer. A strength of the present study is that a questionnaire focusing on shoulder function, namely WOOS, was used in combination with a questionnaire that evaluates back and shoulder function specifically after LD breast reconstruction, the BREAST-Q LD.

There are no previous studies using WOOS to evaluate LD flap transfer. One study^14^, including 35 patients reconstructed with an LD flap and 41 patients reconstructed with an implant, used the Western Ontario Shoulder Instability questionnaire (WOSI). This has partly the same items as WOOS, but there are slight differences^23,40^. The total score for the LD group was 72 per cent and 60 per cent for the controls^14^, which is considerably lower than for the LD group in the present study. Patients with osteoarthritis^29,30^ score substantially lower on WOOS than the LD group of the present study. Using WOOS, more patients in the LD group than in the DIEP control group report subjective discomfort. The clinical significance of the differences seen in this study remain unclear, as the average difference between the groups was about half of the percentage points of the previously calculated MCIDs^29^. This should, however, be interpreted with caution, as the MCIDs have been calculated in patients with glenohumeral osteoarthritis.

BREAST-Q LD has previously been used in two studies^22,26^, but comparison of average measures with those studies are complicated by differences in patient demography and follow-up time. There is a lack of information on the effect on scores of factors such as radiotherapy and axillary surgery in both studies, and they include a mix of delayed and immediate reconstructions. The follow-up in the study of Browne *et al.*^22^ was 18 months and 4 years in the study of Koh *et al.*^26^. The mean scores given (66 and 68, respectively) were slightly higher than the mean score of the LD cases in the present study (63) and closer to that of the DIEP controls (70). It is difficult to estimate the clinical significance of the differences as there are no MCIDs available for BREAST-Q LD^41^. Looking at percentages of patients who have symptoms most/all of the time^22,26^, the frequencies of patients were generally higher in the present study. This could be explained by the considerably different follow-up time.

Previous studies are conflicting regarding long-term effects of LD transfer on back and shoulder function, and many studies conclude that function recovers to almost normal over time^10,11,14^. The present findings contradict this as the patients still have a lower function compared to the controls even 7 years after the operation. There are no long-time longitudinal studies exploring back and shoulder function after LD flaps.

Predictors for a low patient-reported back and shoulder function included axillary surgery and axillary radiotherapy, especially when combined, as well as older age at reconstruction. It might thus be warranted to use LD reconstruction with care in such patients. Pain and musculoskeletal problems increase with age^42,43^, and in the general population one-quarter of adults over the age of 65 report back pain and one-fifth report shoulder pain^42,44^. More studies are thus needed on whether LD flap transfer affects the natural deterioration of back and shoulder function that occurs with age.

## Supplementary Material

znad296_Supplementary_Data

## Data Availability

The participants of this study did not give written consent for their data to be shared publicly, so due to the sensitive nature of the research supporting data are not available.
