# Supplementary material for: Long-term patient-reported back and shoulder function after delayed breast reconstruction with a latissimus dorsi flap: case–control cohort study
Source: Br J Surg. 2023 Oct 25;111(1):znad296. doi: 10.1093/bjs/znad296 (PMC10769156; doi:10.1093/bjs/znad296)
Supplement: znad296_Supplementary_Data [file znad296_supplementary_data.docx]

**Long-term patient-reported back and shoulder function after delayed breast reconstruction with a latissimus dorsi flap– a case-control cohort study with a seven-year follow-up**

Jonas Löfstrand^1,2^, MD, MSc, Anna Paganini^1,2,3^, RN, PhD, Anna Grimby-Ekman^4^, Mattias Lidén^1,2^, MD, PhD, Emma Hansson^1,2^, MD, MA, PhD

1. Department of Plastic Surgery, Institute of Clinical Sciences, The Sahlgrenska Academy, University of Gothenburg, Gröna Stråket 8, SE-413 45 Gothenburg, Sweden
2. Region Västra Götaland, Sahlgrenska University Hospital, Department of Plastic Surgery, Gröna Stråket 8, SE-413 45, Gothenburg, Sweden
3. Institute of Health and Care Sciences, Sahlgrenska Academy, University of Gothenburg, Gothenburg, Sweden
4. School of Public Health and Community Medicine, Institute of Medicine, The Sahlgrenska Academy, University of Gothenburg, Medicinaregatan 18A, 405 30 Gothenburg, Sweden

**Corresponding author.** Jonas Löfstrand, Department of Plastic Surgery, Sahlgrenska University Hospital, Gröna Stråket 8, SE-413 45 Gothenburg, Sweden. Tel: +46313421000/Fax: 031 82 79 03. E-mail: jonas.lofstrand@vgregion.se

**Supplementary Materials**

[Previous studies using BREAST-Q LD and WOOS 3](#_Toc146028932)

[References 6](#_Toc146028933)

# Previous studies using BREAST-Q LD and WOOS

|  | **Patients** | **Follow-up** | **Scores** | **Comments** |  |
| --- | --- | --- | --- | --- | --- |
| **BREAST-Q LD** |  |  |  |  |  |
| Browne, 2018, UK ^1^ | 1096 patients operated with an LD. 180 of them were delayed reconstruction in combination with an implant. | 18 months (all) | Mean 66.3 (SD 18.3).  In group with implants: 67.4 (SD NR)  Frequency of patients who had symptoms most or all of the time:  Back pain: 11%  Shoulder pain: 7%  Shoulder stiffness:7%  Tightness when stretching arm: 19%  Pulling feeling in back: 19%  Weakness in arm: 16%  Difficulty carrying heavy objects: 23%  Difficulty reaching for objects: 21  Difficulty doing activities, arms outstretched: 14%  Difficulty doing activities, arms above head: 13.5  Difficulty, repeat use of shoulder/back muscles: 17.7% | Information on radiation NR. No difference in patients who had complications. |  |
| Koh, 2018, Australia ^2^ | 60 patients  Mean age: 51 (range 32-73) | 4 years (mean) | Mean 68 (95% CI 62-74)  Unilateral (n=46): 65 (60-70)  Bilateral: 70 (63-77)  Delayed or mixed: 69 (61-77)  SNB: 68 (63-77)  Axillary clearance: 62 (56-68) | Scores in mastectomy only group (n=59): 66 (95% CI 62-70) |  |
| **WOOS** (examples) |  |  | Pre-operative total scores | Pre-operative per centage of normal shoulder function |  |
| Baumgarten, 2021, USA ^3^ | N= 1218  Patients undergoing total shoulder arthroplasty |  |  | Mean 36% (SD 17) |  |
| Nyring, 2021, Denmark ^4^ | N=42 (36% male)  Patients with primary glenohumeral osteoarthritis |  |  | Mean: 33% (SD 19, range 5.5-74) |  |

# References

1. Browne JP, Jeevan R, Pusic AL, Klassen AF, Gulliver-Clarke C, Pereira J, Caddy CM, Cano SJ. Measuring the patient perspective on latissimus dorsi donor site outcomes following breast reconstruction. *J Plast Reconstr Aesthet Surg* 2018;**71**(3): 336-343.

2. Koh E, Watson DI, Dean NR. Quality of life and shoulder function after latissimus dorsi breast reconstruction(). *J Plast Reconstr Aesthet Surg* 2018;**71**(9): 1317-1323.

3. Baumgarten KM. The American Shoulder and Elbow Surgeons score has excellent correlation with the Western Ontario Osteoarthritis score. *J Shoulder Elbow Surg* 2021;**30**(11): 2604-2610.

4. Nyring MRK, Olsen BS, Amundsen A, Rasmussen JV. Minimal Clinically Important Differences (MCID) for the Western Ontario Osteoarthritis of the Shoulder Index (WOOS) and the Oxford Shoulder Score (OSS). *Patient Relat Outcome Meas* 2021;**12**: 299-306.
